# Ex Vivo Host Transcriptomics During *Cryptococcus neoformans*, *Cryptococcus gattii*, and *Candida albicans* Infection of Peripheral Blood Mononuclear Cells From South African Volunteers

**DOI:** 10.1093/infdis/jiae410

**Published:** 2024-08-19

**Authors:** Ronan M Doyle, Shichina Kannambath, Alan Pittman, Rene Goliath, Vinod Kumar, Graeme Meintjes, James Milburn, Mihai G Netea, Thomas S Harrison, Joseph N Jarvis, Tihana Bicanic

**Affiliations:** Department of Clinical Research, London School of Hygiene and Tropical Medicine; Genomics Facility, The Institute of Cancer Research; Institute of Infection and Immunity, St George's University London, and Clinical Academic Group in Infection, St George's University Hospitals NHS Foundation Trust, London, United Kingdom; Institute of Infection and Immunity, St George's University London, and Clinical Academic Group in Infection, St George's University Hospitals NHS Foundation Trust, London, United Kingdom; Department of Medicine and Institute of Infectious Disease and Molecular Medicine, University of Cape Town, South Africa; Botswana Harvard Health Partnership, Gaborone; Department of Medicine and Institute of Infectious Disease and Molecular Medicine, University of Cape Town, South Africa; Department of Clinical Research, London School of Hygiene and Tropical Medicine; Botswana Harvard Health Partnership, Gaborone; Department of Internal Medicine and Radboud Center for Infectious Diseases, Radboud University Medical Center, Nijmegen, The Netherlands; Institute of Infection and Immunity, St George's University London, and Clinical Academic Group in Infection, St George's University Hospitals NHS Foundation Trust, London, United Kingdom; Medical Research Council Centre for Medical Mycology, University of Exeter, United Kingdom; Department of Clinical Research, London School of Hygiene and Tropical Medicine; Botswana Harvard Health Partnership, Gaborone; Institute of Infection and Immunity, St George's University London, and Clinical Academic Group in Infection, St George's University Hospitals NHS Foundation Trust, London, United Kingdom

**Keywords:** *Cryptococcus neoformans*, *Cryptococcus gattii*, *Candida albicans*, fungal infection, immune response

## Abstract

*Cryptococcus neoformans*, *Cryptococcus gattii*, and *Candida albicans* are opportunistic fungal pathogens associated with infections in immunocompromised hosts. Cryptococcal meningitis (CM) is the leading fungal cause of human immunodeficiency virus–related deaths globally, with the majority occurring in Africa. The human immune response to *C albicans* infection has been studied extensively in large genomics studies whereas cryptococcal infections, despite their severity, are comparatively understudied. Here we investigated the transcriptional response of immune cells after in vitro stimulation with in vitro *C neoformans*, *C gattii*, and *C albicans* infection of peripheral blood mononuclear cells collected from healthy South African volunteers. We found a lower transcriptional response to cryptococcal stimuli compared to *C albicans* and unique expression signatures from all 3 fungal stimuli. This work provides a starting point for further studies comparing the transcriptional signature of CM in immunocompromised patients, with the goal of identifying biomarkers of disease severity and possible novel treatment targets.

## Teaser

Understanding the host response to infection will be key to generating new antifungals for cryptococcal disease. In this study, we captured the transcriptional signature of cells from South African volunteers when they were exposed to *Cryptococcus neoformans*, *Cryptococcus gattii*, and *Candida albicans*.

*Cryptococcus neoformans* and *Cryptococcus gattii* are ubiquitous environmental organisms to which humans are exposed via inhalation. Invasive infection is rare in immunocompetent hosts; however, in individuals with deficient immunity, dissemination occurs from the lungs into the central nervous system, causing cryptococcal meningitis (CM). CM is one of the leading causes of human immunodeficiency virus (HIV)–related deaths globally, with mortality ranging from 30% to 70%, and >75% of CM cases occur in Africa [1].

Studies of the immune response to cryptococci show pattern recognition of cryptococcal components, such as glucuronoxylomannan and mannoproteins, and activation of CD4^+^ T cells, mainly Th1, leading to activation of M1 macrophages and yeast cell engulfment [2, 3], mediated via the production of the cytokines interferon gamma (IFN-γ), tumor necrosis factor alpha (TNF-α), interleukin (IL) 6, and IL-17, among others [4, 5]. The association of CM with advanced HIV disease, characterized by depletion of CD4^+^ T cells, illustrates the importance of CD4-mediated host defense [6]. Defects in Th1-type immune responses result in production of Th2-mediated alternatively activated macrophages that allow cryptococci to survive and proliferate [7–9]. Our recent genome-wide association study, which included South African participants of African descent with advanced HIV, identified 6 single-nucleotide polymorphisms upstream from CSF1 associated with susceptibility to cryptococcosis [10].

*Candida albicans* is a human gut commensal associated with mucosal and bloodstream infections. It is another opportunistic fungal pathogen in the context of advanced HIV. Compared to *Cryptococcus*, *C albicans* is a well-studied organism, and human studies have used transcriptional profiling to understand the host immune response to infection. *Candida albicans* infections activate both innate immune cells such as neutrophils and macrophages, as well as inducing Th1- and Th17-mediated immune response [11, 12]. While host defense mechanisms mediated by innate immune cells, especially neutrophils, are crucial in systemic *Candida* infections, Th17 responses mainly mediate anti-*Candida* mucosal host defense. During invasion of the tissues, *C albicans* adapts and switches to its filamentous hyphal form, allowing escape and immune evasion [13].

Unlike *C albicans*, immunological studies of *C neoformans* and *C gattii* have been limited to a handful of selected cytokine targets or murine models [4–6, 14–16]. Most of the human immunological studies have been performed in European populations, while very limited data are available from populations in Africa, where the burden of CM-related mortality is greatest. Despite expanded antiretroviral therapy access in high HIV-prevalence African settings leading to reductions in the incidence of CM, mortality remains high. Further improvement of antifungal therapy is necessary to improve outcomes and is reliant on an understanding of the host response to the infection. In this study, we undertook the first bulk transcriptome study of the human peripheral blood mononuclear cell (PBMC) response to cryptococcal infection in healthy South African volunteers.

## MATERIALS AND METHODS

### Healthy Human Volunteer Cohort

Following informed consent, 25 mL of whole blood was drawn from 15 (8 female, 7 male) healthy volunteers (confirmed HIV negative) of self-identified Xhosa ethnicity from Cape Town, South Africa. PBMCs were isolated according to a standard Ficoll-Paque plus (GE HealthCare) protocol, counted and adjusted to 5 × 10^5^ cells/mL, then cultured in RPMI 1640 media supplemented with gentamicin 10 mg/mL, L-glutamine 10 mM, pyruvate 10 mM, and 10% heat-inactivated human serum (Sigma, United Kingdom).

### Ethical Approval

The study was approved by the University of Cape Town Human Research Ethics Committee in 2014 (Ref 721/2014), as an extension of the original human genome-wide association study (Ref 018/2005), published separately [10].

### PBMC Stimulations

A total of 5 × 10^5^ PBMCs were stimulated for 24 hours at 37°C with 3 fungal stimuli: *C neoformans* H99, *C gattii* R265, and *C albicans* UC280, or remained unstimulated. *Cryptococcus neoformans*, *C gattii*, and *C albicans* were cultured on Sabouraud dextrose (SD) agar and incubated for 48 hours at 30°C. Up to 5 colonies were sampled and inoculated in 5 mL of SD liquid media and cultured overnight at 30°C with gentle agitation (165 rpm). Fungal cells (yeast and hyphae mixture for *C albicans*) were collected after centrifugation (200 rpm for 5 minutes) and washed twice in phosphate-buffered saline (PBS). Heat-killed *C neoformans*, *C gattii*, and *C albicans* were prepared by incubating the fungal cells at 65°C for 2 hours and then the cells were washed twice in PBS before stimulation assays. The multiplicity of infection used was 0.1. After washing with PBS, *C neoformans* and *C gattii* cells were opsonized with monoclonal anti-capsule (18B7) antibody (kindly provided by A. Casadevall, Johns Hopkins School of Medicine, Baltimore, Maryland). For the phagocytosis assay, opsonized *C neoformans* and *C gattii* were stained with Calcofluor White (Sigma) for 15 minutes prior to co-culture with PBMCs. In the phagocytosis assay, on average 20%–30% of the CD14^+^ cells were identified with internalized fungus during 6 hours of incubation and 40%–65% during 24 hours of incubation. Cells were harvested for RNA extraction at 6 and 24 hours from unstimulated and stimulated PBMCs using TRIzol (Life Technologies) reagent according to the manufacturer's instructions and treated with DNase to ensure elimination of genomic DNA. RNA concentration and quality were analyzed using a Qubit fluorometer (Life Technologies) and 2200 Tape station (Agilent Technologies), respectively.

### RNA Sequencing and Analysis

RNA sequencing libraries were prepared using the TruSeq RNA Library Preparation Kit v2 (Illumina), according to the manufacturer's instructions. Libraries were then sequenced to generate 150-bp paired-end reads on an Illumina NovaSeq 6000 using an S4 flow cell. Read quality was assessed using FASTQC (https://www.bioinformatics.babraham.ac.uk/projects/fastqc/) and samples with too few reads prior to alignment were removed using fastp software (version 0.20.0) (https://github.com/OpenGene/fastp). Sequences were aligned to the human reference genome (hg38) with STAR (version 2.6.8a) [17], and samples with a low percentage of reads mapped to the reference were removed. Differential gene expression was performed using DESeq2 (v1.30.1) [18] in R (v3.8.3). Gene Ontology (GO) analysis was carried out using goseq (v1.54.0) [19], also in R. Pathway analysis was carried out using Reactome [20].

## Supplementary Material

jiae410_Supplementary_Data

## Figures and Tables

**Figure 1.**
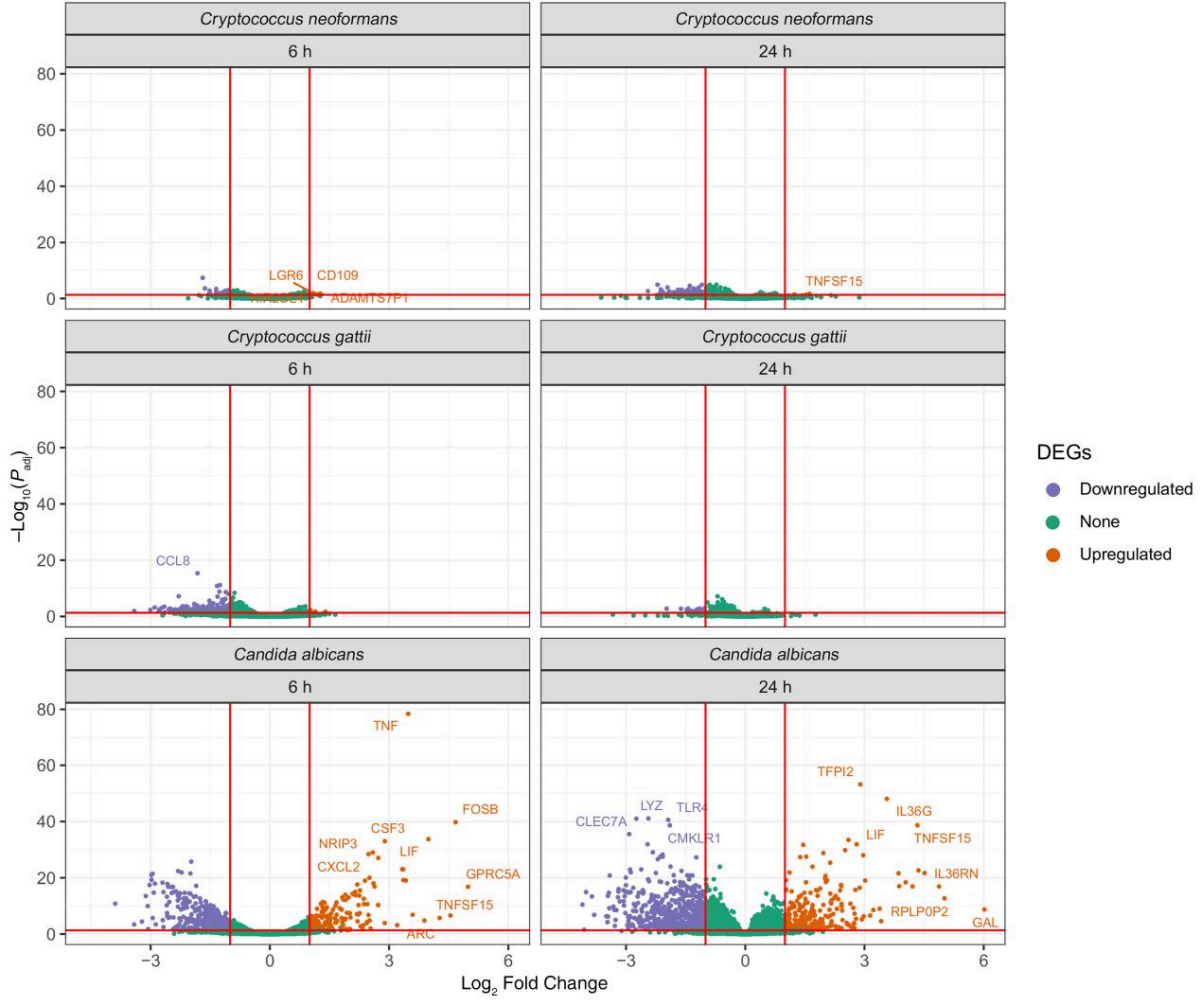
Volcano plots showing the number of differentially expressed genes (DEGs) when compared to unstimulated controls for *Cryptococcus neoformans*, *Cryptococcus gattii*, and *Candida albicans* at 6 h and at 24 h.

**Figure 2.**
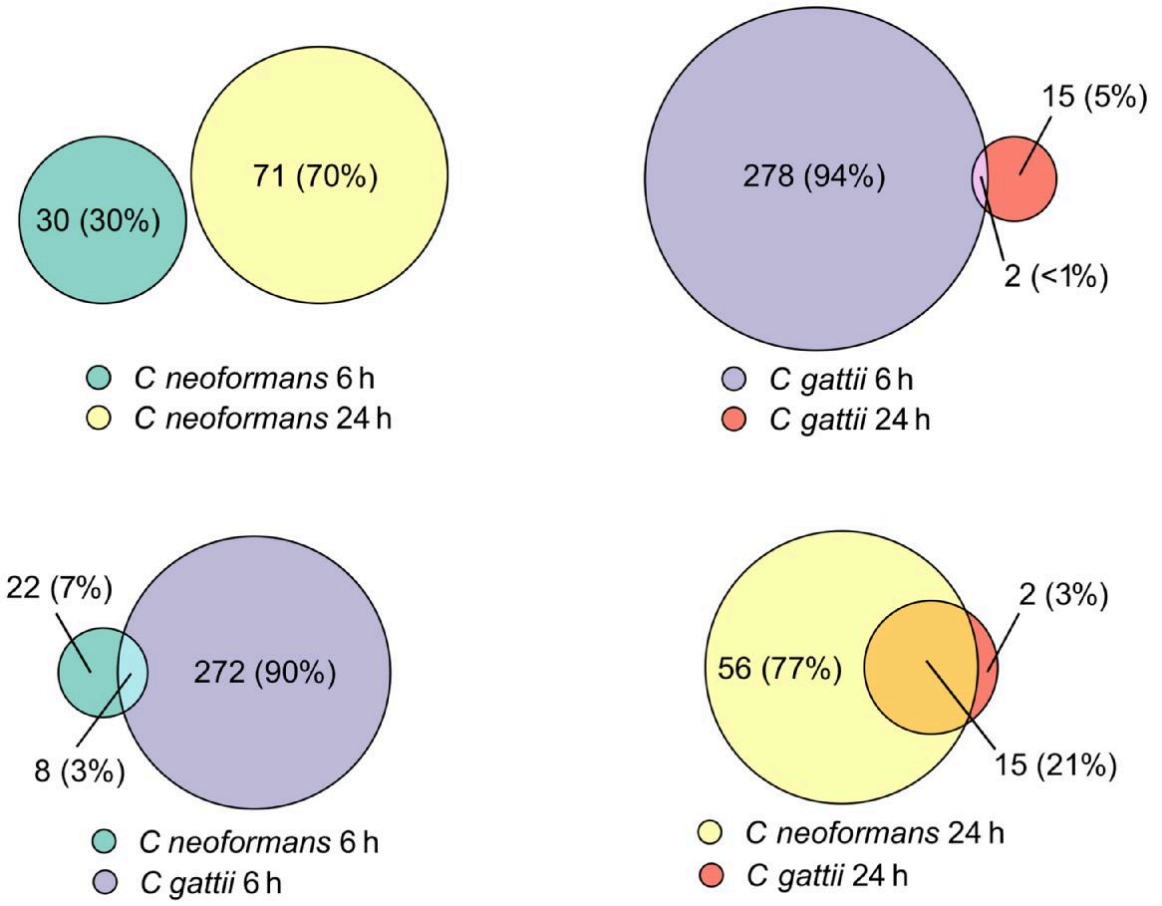
Euler diagram showing number of differentially expressed genes shared between *Cryptococcus neoformans* at 6 h and 24 h, and the same comparison between *C neoformans* and *Cryptococcus gattii* at the same time points.

**Figure 3.**
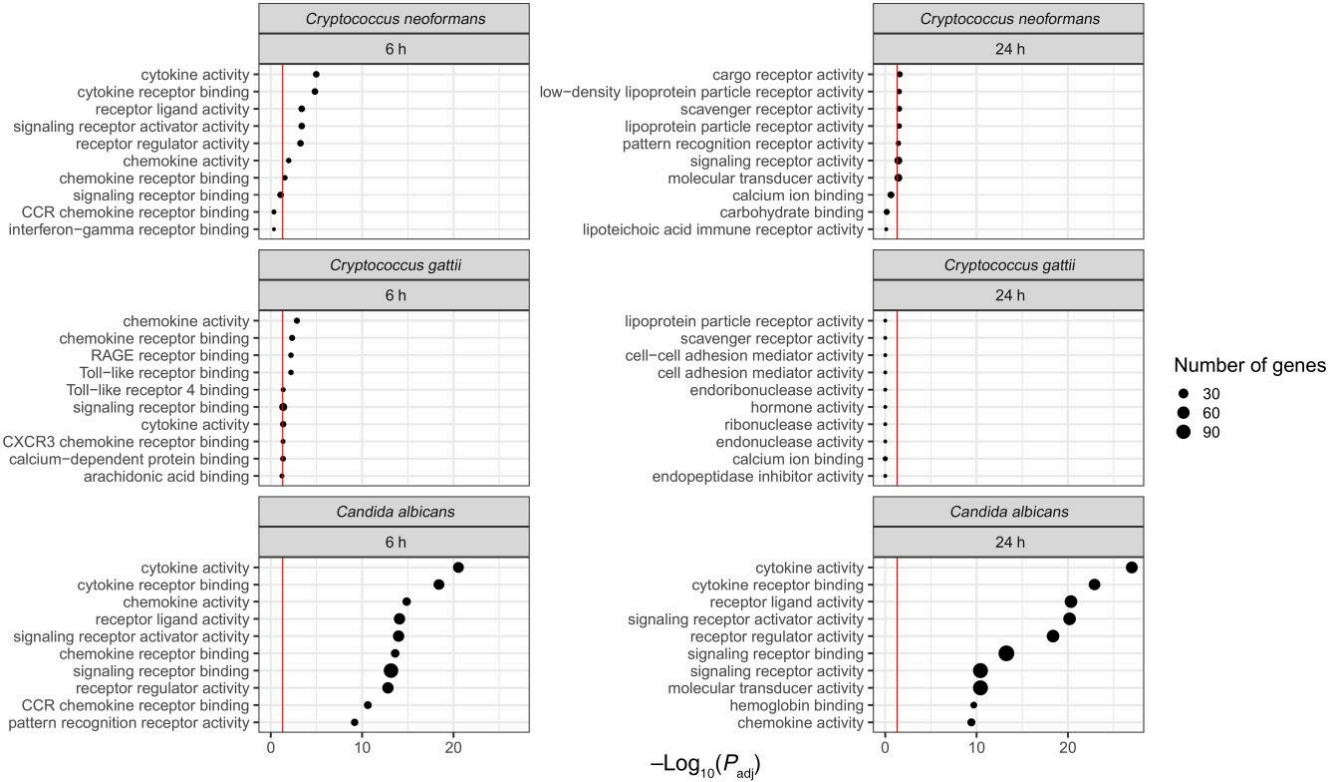
Gene Ontology molecular function pathways enriched in *Cryptococcus neoformans*, *Cryptococcus gattii*, and *Candida albicans* at 6 and 24 h poststimulation. Top 10 pathways displayed by lowest adjusted *P* value; red line denotes adjusted *P* value <.05.

**Figure 4.**
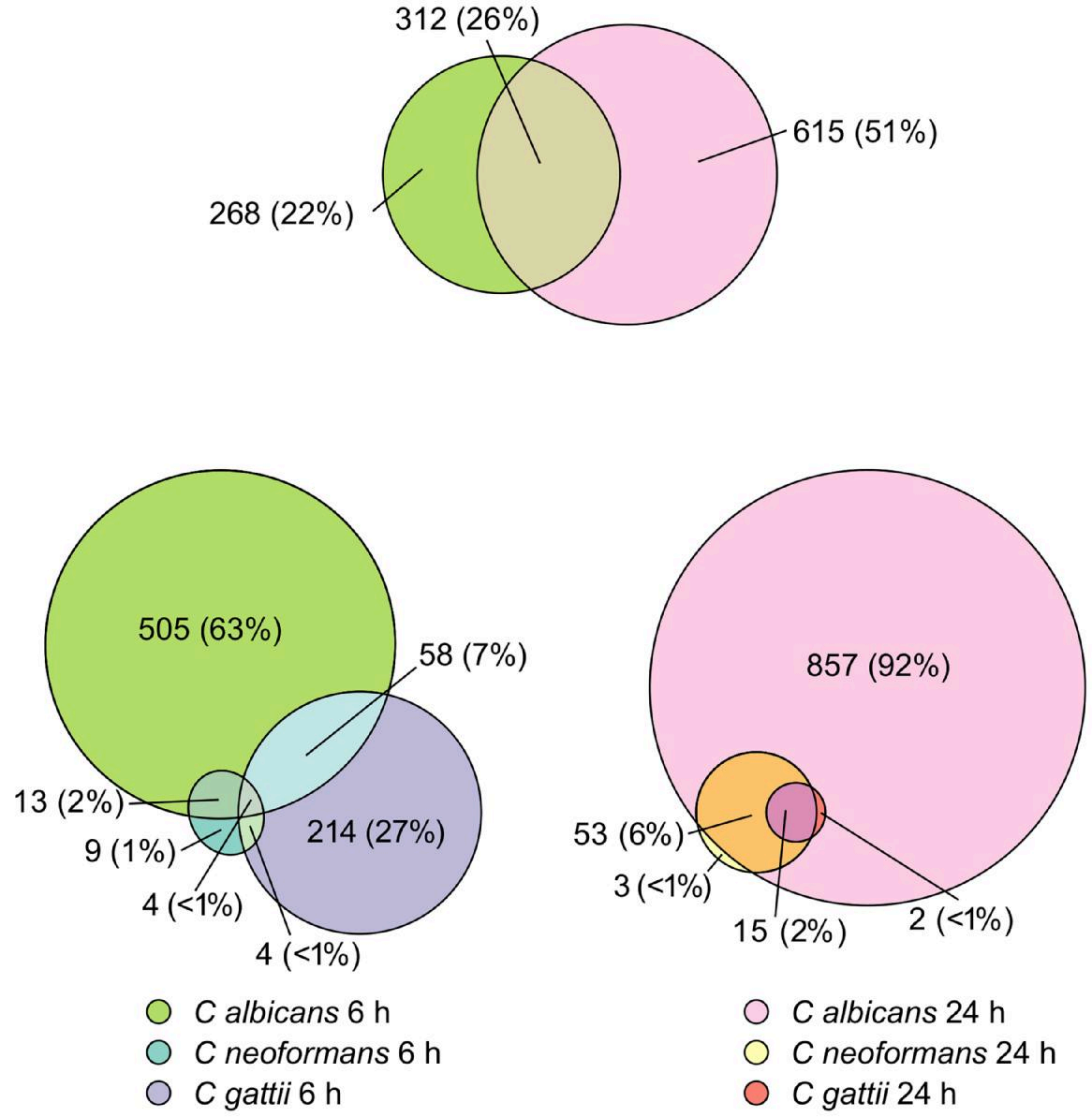
Euler diagram showing number of differentially expressed genes shared between *Candida albicans* at 2 different time points, and the same comparison between *Cryptococcus neoformans*, *Cryptococcus gattii*, and *C albicans* at both 6 h and 24 h poststimulation.

**Figure 5.**
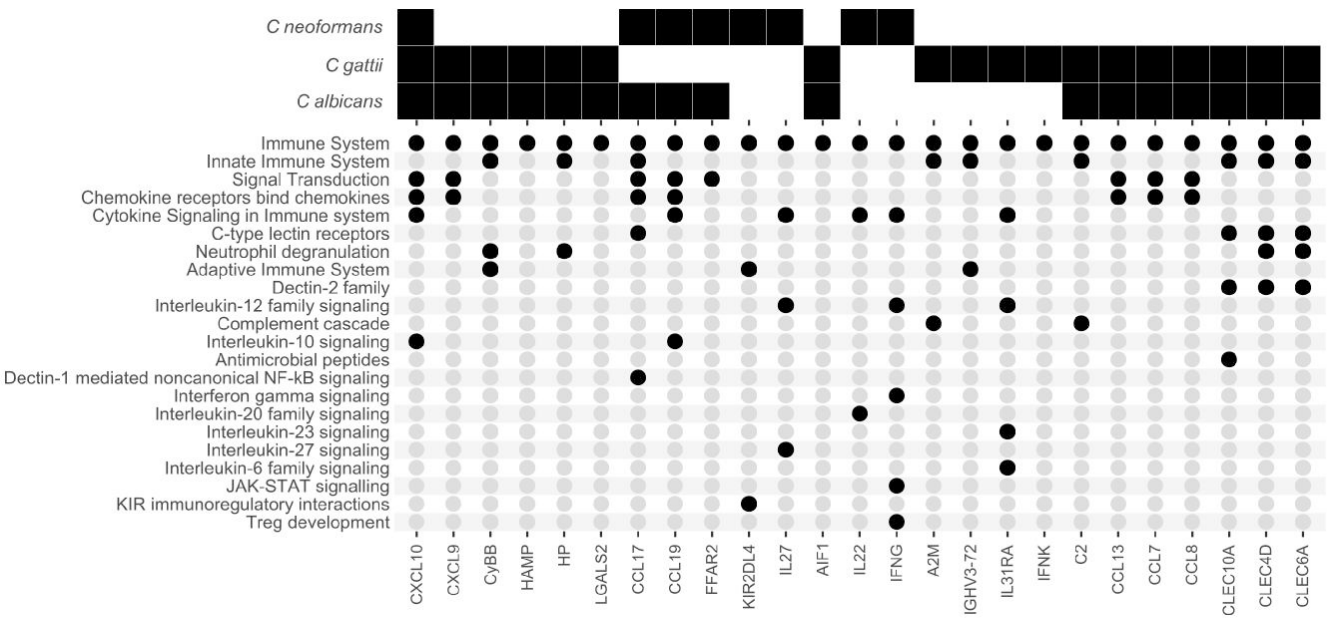
Expression of proinflammatory genes following heat-killed *Cryptococcus neoformans*, *Cryptococcus gattii*, and *Candida albicans* stimulation. A solid black square denotes a gene that is significantly differentially expressed in specific pathogen samples when compared to unstimulated controls. Black circles correspond to the Reactome immunological pathways each gene is associated with.

## Data Availability

The raw RNA sequencing data produced during this study are split over 2 repositories. The data are available from figshare via https://figshare.com/s/b953f3192c77cef0be98 and the European Nucleotide Archive under study accession number PRJEB74561.

## RESULTS

### Cryptococcal-Induced Differential Gene Expression in Human PBMCs

To understand the transcriptional response to cryptococcal stimulation, we compared PBMCs from 15 healthy donors stimulated with 2 cryptococcal species commonly causing human CM (*C neoformans* and *C gattii*) with unstimulated cells. RNA was isolated from PBMCs at 6 and 24 hours poststimulation, and mRNA was then sequenced from each sample, generating a mean of 36.6 million reads per sample. We determined the differentially expressed genes (DEGs) at both time points using a cutoff of >1 log_2_ fold change in expression and Benjamini-Hochberg–adjusted *P* < .05. *Cryptococcus gattii* had the greater number of DEGs at 6 hours poststimulation (280 vs 30), whereas *C neoformans* had more abundant DEGs at 24 hours poststimulation (71 vs 17) (Figure 1). There were 5 upregulated and 25 downregulated DEGs for *C neoformans* at 6 hours, and 3 upregulated and 68 downregulated DEGs after 24 hours. For *C gattii* we found 4 upregulated and 276 downregulated DEGs at 6 hours and 0 upregulated and 17 downregulated DEGs after 24 hours. The full list of DEGs associated with *C neoformans* and *C gattii* stimulation at both time points can be found in Supplementary Table 1.

We compared the fraction of shared DEGs between the 2 time points for both pathogens and found little (<1%) to no crossover between gene expression within species at the 2 time points (Figure 2). *Cryptococcus neoformans* shared no DEGs between the 6- and 24-hour time points, whereas *C gattii* shared 2 DEGs between 6 and 24 hours, one of which was an RNA pseudogene (*RN7SL4P*) and the other a long intergenic nonprotein coding RNA (lncRNA) gene (*LINC01094*).

There were also only a minority of shared DEGs between species at the same time points; 3% (8 genes) of total DEGs were shared at 6 hours, and this rose to 21% (15 genes) of DEGs at 24 hours.

### Cryptococcal Pathway Analysis

We used GO terms to functionally annotate these DEGs found poststimulation with *C neoformans* and *C gattii*. Analyzing the GO molecular function pathways at 6 hours poststimulation showed that *C neoformans* had 7 pathways significantly altered compared to unstimulated controls, whereas *C gattii* had 13 (Figure 3). Both pathogens had significantly enriched proinflammatory pathways containing genes associated with cytokine and chemokine activity and receptor binding. GO molecular function pathways significantly differentially expressed in *C gattii* at 6 hours poststimulation included Toll-like receptors and CXCR3 chemokine receptors. GO biological processes significantly associated with *C gattii* included neutrophil chemotaxis and genes associated with a cell defense response to fungi. GO biological processes significantly associated with *C neoformans* 6 hours poststimulation included proinflammatory pathways associated with T-cell activation and differentiation (Supplementary Figure 1). By 24 hours, none of these 6-hour proinflammatory pathways remained significantly enriched for either cryptococcal stimulus.

### *Candida albicans–*Induced Differential Gene Expression in Human PBMCs

We compared cryptococcal responses to *C albicans* as a model for a previously well-characterized antifungal immune response. As described previously, we stimulated PBMCs from the same 15 healthy donors with heat-killed *C albicans* and isolated RNA at 6 and 24 hours. When compared to the heat-killed cryptococcal species, a much more pronounced inflammatory response was seen in *C albicans*–stimulated cells at the same time points. There were 580 DEGs at 6 hours poststimulation with *C albicans* and 927 at 24 hours, compared to 280 and 30 at 6 hours and 17 and 71 at 24 hours for *C gattii* and *C neoformans*, respectively. For *C albicans* DEGs, this corresponded to 413 downregulated and 167 upregulated at 6 hours and 656 upregulated and 271 downregulated at 24 hours. The full list of DEGs associated with *C albicans* stimulation at both time points can be found in Supplementary Table 1.

When we compared the fraction of shared DEGs for *C albicans* between 6 and 24 hours, we found that 26% (Figure 4) of the total number of DEGs were shared between both time points, compared to the completely distinct cryptococcal responses at 6 and 24 hours. At 24 hours poststimulation, almost the entirety of the transcriptional response found in *C neoformans–* and *C gattii–*stimulated cells matched with the DEGs found in *C albicans–*stimulated cells (70/73 DEGs [96%]). However, as noted above, the overall scale of the response from exposure to *C albicans* was greater than that of the cryptococcal stimuli (Figure 1).

As with the cryptococcal stimuli, we used GO terms to functionally annotate the DEGs found poststimulation with *C albicans* (Figure 3). Similar to *C neoformans* and *C gattii*, proinflammatory genes within cytokine, chemokine, and receptor binding pathways were significantly enriched. However, the scale of enriched pathways was greater than the other cryptococcal stimuli and included additional pathways such as CCR chemokine receptors and immunoglobulin G binding. Comparing GO biological process pathways, at 6 hours, among the proinflammatory pathways significantly enriched were those associated with myeloid leukocyte migration, whereas by 24 hours this was replaced by cytokine production and its regulation.

### Comparative Host Transcriptomics Between Cryptococcal and *C albicans* Stimulations

We further wanted to determine whether there were unique features of cryptococcal inflammatory responses compared to those following *Candida* stimulation, as well as any differences between *C neoformans* and *C gattii*. As previously noted, no inflammatory pathways were significantly altered at 24 hours post–cryptococcal stimulation. We therefore focused on the 6 hours poststimulation timepoint, as this comprised proinflammatory signatures common to all 3 species (Figure 3 and  Figure 5). There were 505 unique DEGs associated with *C albicans* stimulation, 214 associated with *C gattii* stimulation, and only 6 unique DEGs associated with *C neoformans* stimulation (Figure 3). To determine the role of this group of genes, we again functionally annotated them using GO terms as above, retaining only genes that had a higher order GO biological process annotation corresponding to “immune system process.” We used Reactome pathway analysis to group these genes into specific inflammatory pathways (Figure 5). We found only a single proinflammatory DEG associated with all 3 pathogens, *CXCL10*, a chemokine strongly induced by IFN-γ and recognized as a component of the IL-10 signaling pathway in the Reactome pathway database. *Candida albicans* and *C gattii* shared the highest number of inflammatory DEGs (15 DEGs) compared to *C neoformans* and *C albicans* (4 DEGs) and we found no DEGs shared solely between *C neoformans* and *C gattii*. Common pathways shared between *C albicans* and *C gattii* included Dectin-2 C-type lectin receptors and the complement cascade, all of which are important elements of the innate immune response to these fungi. Pathways enriched uniquely following *C neoformans* stimulation included those associated with killer immunoglobulin-like receptors and regulatory T cells.

## DISCUSSION

In this study we describe for the first time the transcriptome response to stimulation of human PBMCs by commensal and environmental fungal pathogens in healthy South African volunteers, and we show unique gene expression signatures in *C neoformans*, *C gattii*, and *C albicans*. The scale of the transcriptional response following stimulation by both cryptococcal species was considerably smaller than that to *C albicans*. The cryptococcal inflammatory response peaked at 6 hours and had subsided by 24 hours, whereas the *C albicans* response persisted at 24 hours poststimulation.

There are many possible reasons for these differences. *Candida albicans* is a human commensal of the gastrointestinal and vulvovaginal tracts causing mucosal or bloodstream infections in advanced HIV disease and invasive disease (candidiasis or candidemia) in hospitalized patients, often in the context of mucosal barrier breach [21], whereas *Cryptococcus* is an environmental saprophyte acquired by inhalation, causing invasive infection (CM) largely in immunocompromised hosts. Human immune responses to *Cryptococcus* are triggered by phagocytosis of encapsulated cryptococcal cells (enhanced by opsonizing antibodies and complement) by macrophages/dendritic cells and subsequent antigen presentation to T cells (CD4^+^, CD8^+^, and natural killer [NK] cells), orchestrated and potentiated by proinflammatory cytokines such as TNF-α and IFN-γ. The capsule is anti-phagocytic and disrupts T-cell proliferation, which could explain the paucity of inflammatory responses seen in our study. Of the 2 cryptococcal pathogens, *C gattii* is responsible for relatively more disease in immunocompetent hosts [22, 23], and this seems to corroborate with the more pronounced inflammatory response when compared to *C neoformans*, as previously shown.

There has not been a prior study of the human transcriptome response to fungal pathogens from immunocompetent African volunteers. A study in China found similar upregulated pathways, such as NF-kB signaling and JAK-STAT pathway signaling, following heat-killed *C neoformans* stimulation of 15 healthy volunteers [24]. We found little overlap in the individual DEGs between these 2 studies, which may underline differences in either host- or pathogen-related factors, as well as methodological differences (the Chinese study used monocytes alone and a different timepoint of 12 hours).

The inflammatory response to *C albicans* was of a greater magnitude compared to *C neoformans* and *C gattii* and included some overlap in DEGs. However, there was only a single inflammatory Reactome pathway overrepresented in all 3 fungal species, which was IL-10 signaling through the differential expression of *CXCL10*. This has previously been identified as a protective or at least secondary response to regulate inflammation in all 3 pathogens [5, 11, 25, 26]. Other than this pathway, the inflammatory response caused by heat-killed *C neoformans* and *C gattii* seem to be distinct from each other. A transcriptomics study of mice infected with *C neoformans* and *C gattii* also identified distinct gene expression signatures caused by the 2 fungal species, but they were not entirely unique [27]. Although there were differences, the study also found classical complement activation as unique to *C gattii* infection, compared to *C neoformans*, and expression of NK cell genes, such as receptor *KIR2DL4*, unique to *C neoformans.*

Another significantly expressed gene unique to *C neoformans* stimulation was IFN-γ, which we and others have previously described as key in the clearance of infection [4, 28]. *IL27* was also significantly differentially expressed in *C neoformans* samples and has been associated with the well-characterized cryptococcal immune reconstitution inflammatory syndrome [28].

A feature common to all the fungal stimuli includes innate immune recognition of fungal cell wall antigens. We identified shared pathways between *C neoformans* and *C albicans* that included Dectin-1–mediated NF-kB signaling; between *C gattii* and *C albicans* we identified enhanced expression of genes encoding for Dectin-2 family signaling. For *C albicans*, important innate immune pattern recognition receptors are C-type lectin receptors (CLRs) of the Dectin-1 cluster that recognize the (1,3)-β-D-glucans in its cell wall [29]. Dectin-2 is another CLR that recognizes the α-mannan constituent of the *C albicans* cell wall [30] and has also been previously shown to bind the same proteins in the *C gattii* cell wall [31].

Limitations of this study include the relatively small number of healthy controls and a lack of comparison to a diseased cohort. In this study, while we have attempted to recreate real-world infection conditions ex vivo, these are not going to be identical to a genuine infection. For example, using heat-killed fungi, opsonizing with monoclonal antibodies and multiplicity of infection all introduce possible variables that are not present in invasive human infections, but these are nevertheless consistent with other published studies using *Candida* [11, 32, 33] and *Cryptococcus* [24, 34–37] and are necessary in order to carry out temporal studies on human cells. Unfortunately for these healthy volunteers, we do not have clinical or serological data to ascertain prior exposure to any of the pathogens included in this study. However, given the commensalism of *C albicans* and the high seroprevalence of cryptococcal antibodies in adult cohorts in the United States [38, 39], we would also expect a high seroprevalence for all fungi in this cohort. There is a major issue in the lack of serological data across southern Africa, given it has the largest the burden of disease, reaffirming the need of studies such as this that focus on a healthy South African population. Further studies quantifying seroprevalence and immune responses to a range of cryptococcal stimuli in southern African populations would be especially valuable. Also, future studies could undertake RNA sequencing from immune cells from blood or cerebrospinal fluid collected from immunocompetent patients as well as those with HIV with a range of CD4 counts, to identify differential gene expression associated with a range of opportunistic pathogens common to this specific patient population, such as *Mycobacterium tuberculosis*, *Pneumocystis jirovecii*, *Candida* spp, and *Cryptococcus* spp.

In conclusion, this study used de novo transcriptomic sequencing to identify unique gene expression signatures that differentiate *C neoformans* and *C gattii* infection from each other, and from *C albicans* infection, in a healthy African control population. This work provides a foundation for further transcriptomic studies to investigate the gene expression signature associated with mortality in CM and provide new insights into potential novel treatment targets.

## Supplementary Data

Supplementary materials are available at *The Journal of Infectious Diseases* online (http://jid.oxfordjournals.org/). Supplementary materials consist of data provided by the author that are published to benefit the reader. The posted materials are not copyedited. The contents of all supplementary data are the sole responsibility of the authors. Questions or messages regarding errors should be addressed to the author.

## Notes

***Acknowledgments.*** We thank the volunteers who participated in the study; nurses at Site B Khayelitsha who organized recruitment and consent for the study; and Professor Robert Wilkinson, University of Cape Town, in whose laboratory the PBMC stimulation and RNA extractions were conducted.

***Disclaimer.*** The views expressed in this publication are those of the authors and not necessarily those of the National Institute for Health and Care Research or the UK Department of Health and Social Care.

***Financial support.*** This work was funded by the National Institute for Health and Care Research through a Global Health Research Professorship to J. N. J. (RP-2017–08-ST2-012) using aid from the UK government to support global health research, and by a Wellcome Trust Strategic Award for Medical Mycology and Fungal Immunology 097377/Z/11/Z, via a postdoctoral fellowship award to T. B. and M. G. N.
