# Supplementary figures and images for: Ex Vivo Host Transcriptomics During Cryptococcus neoformans, Cryptococcus gattii, and Candida albicans Infection of Peripheral Blood Mononuclear Cells From South African Volunteers
Source: J Infect Dis. 2024 Aug 19;231(1):e254–62. doi: 10.1093/infdis/jiae410 (PMC11793030; doi:10.1093/infdis/jiae410)

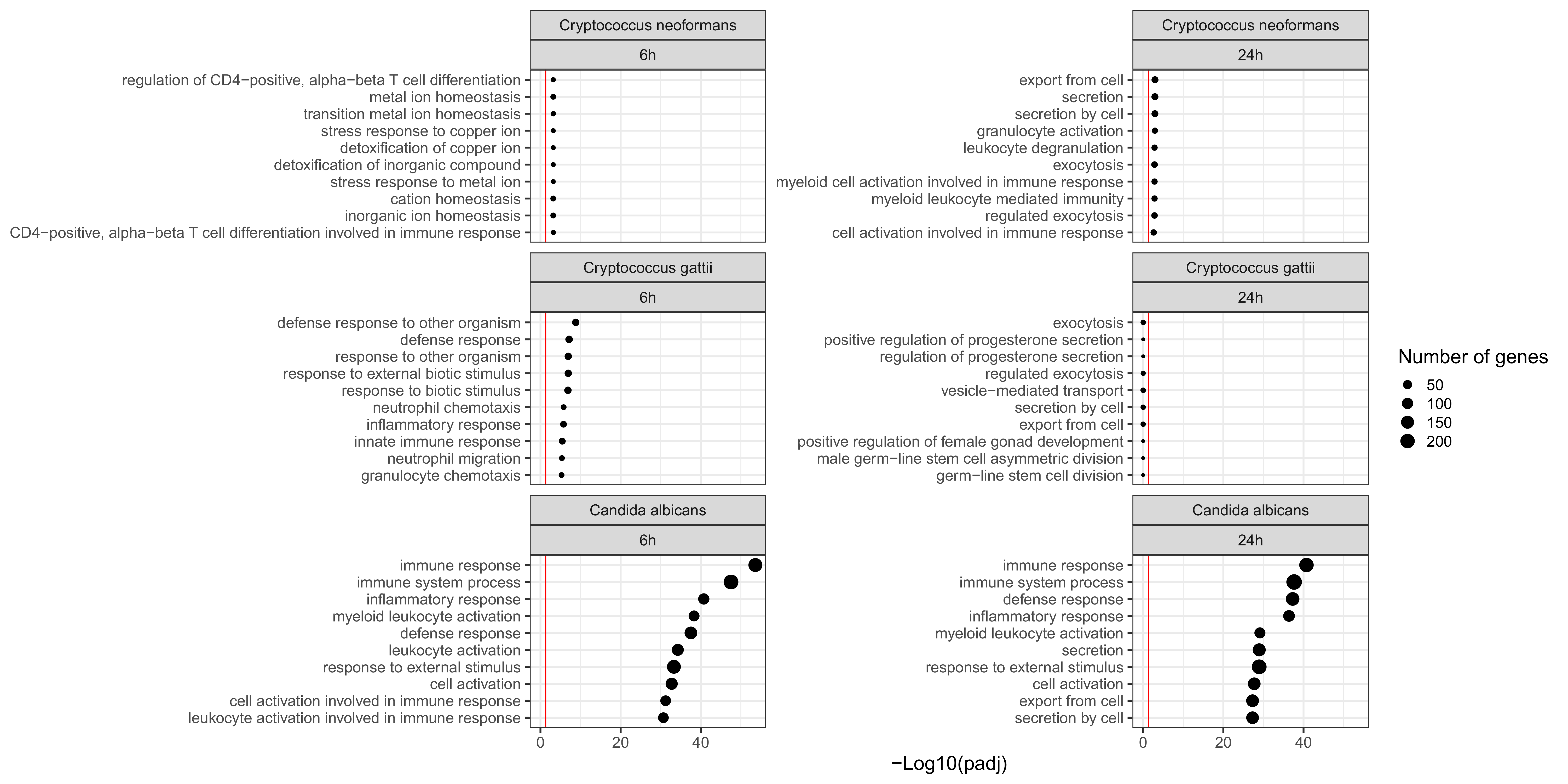

Supplement: jiae410_Supplementary_Data [file jiae410_supplementary_data.zip › supplementary_figure_1.tif]
